# Anti-Inflammatory Effects of *Limosilactobacillus fermentum* KGC1601 Isolated from *Panax ginseng* and Its Probiotic Characteristics

**DOI:** 10.3390/foods11121707

**Published:** 2022-06-10

**Authors:** Heejin Kim, Yun-Seok Lee, Hye-Young Yu, Mijin Kwon, Ki-Kwang Kim, Gyo In, Soon-Ki Hong, Sang-Kyu Kim

**Affiliations:** 1 Laboratory of Red Ginseng Products, Korea Ginseng Corporation, 30, Gajeong-ro, Shinseong-dong, Yuseong-gu, Daejeon 34128, Korea; kiheji1117@naver.com (H.K.); yunseok@kgc.co.kr (Y.-S.L.); hyu@kgc.co.kr (H.-Y.Y.); 20220036@kgc.co.kr (M.K.); 20109042@kgc.co.kr (G.I.); 2 Department of Environmental Engineering, Chungnam National University, Daehak-ro, Yuseong-gu, Daejeon 34134, Korea; kimkk@cnu.ac.kr; 3 Laboratory of Efficacy Research, Korea Ginseng Corporation, 30, Gajeong-ro, Shinseong-dong, Yuseong-gu, Daejeon 34128, Korea; skhong@kgc.co.kr

**Keywords:** anti-inflammatory, lactic acid bacteria, *Limosilactobacillus fermentum*, *Panax ginseng*, probiotics

## Abstract

We investigated the potential probiotic properties of *Limosilactobacillus fermentum* KGC1601 isolated from *Panax ginseng*. Ginseng cultivated in an experimental field of the Korea Ginseng Research Institute was fermented, followed by single colony selection from MRS agar. We performed 16s-rRNA sequencing and whole-genome analysis to identify *L. fermentum* and evaluate the biosafety parameters of this strain, respectively. We confirmed this strain was susceptible to six antibiotics, as proposed by the European Food Safety Authority, did not produce biogenic amines, and did not exhibit any hemolytic activity. Acid resistance and bile salt tolerance, which are essential properties of a probiotic agent, were investigated. Notably, distinguishing properties of this strain were that it exhibited excellent bile salt tolerance and anti-inflammatory effects. The excellent bile salt tolerance was confirmed by scanning electron microscopy. Through qRT-PCR and ELISA studies, it was revealed that *L. fermentum* KGC1601 pre-treatment up-regulates anti-inflammatory cytokines and down-regulates pro-inflammatory cytokines in RAW 264.7 cells. Consequently, we suggested that *L. fermentum* KGC1601 can be safely used as a potential anti-inflammatory functional probiotic agent.

## 1. Introduction

There are more than 100 trillion intestinal bacteria inhabiting the human intestine. Among them, there are several bacteria that play a beneficial role in the host, while there are harmful bacteria that cause various side effects. Maintaining the balance of these strains in the gut microbiome affects human health [1]. Probiotics are defined as “live microorganisms that have beneficial effects on health by maintaining the balance of the host’s intestinal microbiome” [2]. The most common microorganisms used as probiotics include *Lactobacillus* and *Bifidobacterium*, and some *Enterococcus* and *Streptococcus* are also considered probiotics. In particular, *Lactobacillus* and *Bifidobacterium* strains are widely used in fermented milk and functional foods [3]. Although probiotics play an important role in regulating the intestinal microbiome of the human body and contribute to health, in order for microorganisms to be used as probiotics, it is necessary to verify the safety of ingestion and the stability of the digestive system.

Endogenous microorganisms, which internally colonize plant tissues without harming the host plant, can produce secondary metabolites that promote plant growth, control plant pathogens, and enhance plant tolerance to biotic or abiotic stresses [4,5]. Ginseng is the plants belonging to the genus *Panax* and contains useful substances for the human body, including ginsenosides, and is used as a medicinal herb all over the world. Similar to many other plants, ginseng plant contains a wide range of endophytes. Various endogenous bacteria and fungi have been found in all parts of the ginseng plant, and the major genera among them were *Bacillus*, *Pseudomonas*, and *Paenibacillus* [6]. Several studies have identified various bacterial endophytes in *Panax* species. Hong et al. found bacterial endophytes, including Proteobacteria, Actinobacteria, Firmicutes, and Bacteroidetes, in the root of *Panax ginseng* through 16S-rDNA sequencing [7]. Chowdury et al. also reported that similar types of endogenous bacteria were found in *Panax ginseng* roots, stems, and leaves using isolation [8]. Many studies on the endophytes of ginseng have reported the benefits of bacterial and fungal endophytes of ginseng, such as improving plant nutrition, promoting growth, and improving disease resistance. In a recent study by Mo et al., they isolated endophytic *Lactobacillus paracasei* from ginseng and investigated the physiological properties of this strain [9].

*Limosilactobacillus fermentum* is a representative species of the genus *Lactobacillus* that functions as a probiotic. Some *L. fermentum* stains are widely regarded as a safe probiotic strain and have been used as a food fermenter in various industries for a long time. As a probiotic, *L. fermentum* typically possesses acid/bile tolerance and intestinal adhesion activities. Several research papers have reported the health beneficial effects of *L. fermentum*, such as anti-obesity [10,11], cholesterol-lowering effects [12], and immunomodulatory activity [13].

Inflammation is an immune-mediated response to protect the body from the attack of external viruses and pathogenic infectious agents [14]. This response is regulated by pro-inflammatory mediators such as inducible nitric oxide synthase (iNOS) and cyclooxygenase-2 (COX-2) and cytokines such as tumor necrosis factor-α (TNF-α), interleukin-1β (IL-1β), and interleukin-6 (IL-6), which lead to the recruitment of immune cells and induction of systemic responses [15]. Particularly, intestinal inflammation can cause the development of acute symptoms, such as diarrhea, gastric bleeding, and abdominal pain, as well as chronic symptoms, such as inflammatory bowel disease and colorectal cancer [16]. Thus, relative to any other function, the anti-inflammatory effect of probiotics toward improving intestinal health has been continuously studied for a long time. For example, *Lactobacillus plantarum* IDCC 3501 isolated from kimchi exhibited the anti-inflammatory effect through the deregulation of genes associated with pro-inflammatory mediators and cytokines in macrophage cells [17]. In another study, several strains isolated from canine and feline feces were demonstrated to modulate anti-inflammatory activity through the inhibition of iNOS and COX-2 expression [18].

In the present study, we isolated *L. fermentum* strain from *Panax ginseng* for the first time, and evaluated the probiotic potential and the safety of this strain. As basic properties of probiotics, acid resistance, bile salt tolerance, and adhesion ability were investigated. For safety assement as probiotics, hemolytic activity, carbohydrate fermentation, antibiotic resistance, and biogenic amine production were investigated. In addition, we investigated the anti-inflammatory effect of *L. fermentum* KGC1601 as a functional characteristic of this strain.

## 2. Materials and Methods

### 2.1. L. fermentum KGC1601 and Other Bacterial Strains

We isolated endophytic lactic acid bacteria from 6-year-old ginseng (*Panax ginseng* C.A. Meyer) collected from Anseong, Korea. The collected ginseng was washed thoroughly with purified water and then sterilized with 70% ethanol for 1 min. The sample was sliced with a sterile razor blade; then, the sliced samples were mixed with 100 mL of sugar solution containing 1.25% (*w/v*) sucrose and 2.5% (*w/v*) glucose. The mixture was fermented at 37 °C for 5 days; then, the fermented solution was serially diluted in 9 mL sterile saline, and the diluted samples were spread on MRS medium (BD Difco, Frankilin Lakes, NJ, USA) containing 0.5% (*w/v*) CaCO_3_. The plates were incubated at 37 °C for 24–48 h. We selected candidate colonies based on the presence of a transparent halo around colony. One of these colonies was identified as *L. fermentum* through 16s-ribosamal RNA (16s-rRNA) sequencing (Solgent, Korea). We designated this strain as *L. fermentum* KGC1601 and deposited it in the Korea Collection for Type Culture (KCTC, Jungeup, Korea) under the accession number KCTC14731BP.

*L. fermentum* KCTC3112, and *Escherichia coli* KCTC2441 were distributed from KCTC (KCTC, Jungeup, Korea). *L. fermentum* IDCC3901 was distributed from Ildong Bioscience (Pyeongtaek-si, Gyeonggi-do, Korea). *Staphylococcus aureus NCTC10788* was purchased at Bioball^®^ from bioMérieux (Marcy-l’Etoile, France).

### 2.2. L. fermentum KGC1601 Culture and Exopolysaccharide Purification

The strain was cultured in MRS broth and stored in 25% (*w/w*) glycerol at −80 °C. Prior to every experiment, the strain was pre-cultured overnight at 37 °C in MRS broth. We generated cell-free supernatants by inoculating 10% (*v/v*) pre-cultured *L. fermentum* KGC1601 into fresh MRS medium and incubating this overnight at 37 °C. After incubation, the supernatant was collected by centrifugation at 10,000 rpm for 10 min at 4 °C followed by filtration through a 0.22 µm-pore-size membrane filter. The collected supernatant was freeze-dried and stored at −80 °C until used in experiments.

Exopolysaccharide (EPS) was purified using the ethanol precipitation method as previously described [19]. In summary, *L. fermentum* KGC1601-cultured media was separated via centrifugation at 10,000 rpm for 20 min. Media supernatant was then isolated, and trichloroacetic acid was added to denature proteins in the media supernatant at 37 °C for 1 h. Denatured proteins were isolated from the media by centrifugation at 10,000 rpm for 20 min, followed by mixing with absolute ethanol. The separated precipitates were dialyzed with the distilled water at 4 °C for 24–48 h to fully remove the components of media and other substances. The dialyzed solution was then lyophilized to obtain EPS, which was resuspended in distilled water for subsequent experiments and stored at −80 °C.

### 2.3. Whole-Genome Sequencing and Identification of Antibiotic Resistance and Virulence Genes

The whole genome of *L. fermentum* KGC1601 was sequenced on the Illumina Miseq 300 system with 2 × 300 bp paired-end reads using the 600 cycle (Miseq Reagent Kit v3) sequencing kit (Chunlab Inc., Seoul, Korea). Putative virulence factors were identified using Virulence factor database (VFDB; version 2020.02.13; http://www.mgc.ac.cn/VFs/ (accessed on 1 December 2021)). For detection of acquired antimicrobial resistance genes, the assembled sequences were compared with the reference sequences in the ResFinder database (https://cge.cbs.dtu.dk/servies/ResFinder (accessed on 1 December 2021)) using ResFinder v.3.2.

### 2.4. Carbohydrate Fermentation Patterns

The carbohydrate fermentation patterns of the strains were investigated using the API 50 CHL/CH kit (bioMérieux, Marcy-l’Etoile, France) and 49 selected carbohydrate sources according to the manufacturer’s guidelines. The strain was cultured in MRS medium overnight at 37 °C, resuspended in 10 mL of API 50 CHL medium, and then loaded into the API 50CH test strip. Carbohydrate utilization patterns were determined according to the manufacturer’s color reaction chart after incubating the test strips for 48 h at 37 °C.

### 2.5. Determination of Antibiotic Resistance

Antimicrobial susceptibility was tested on 8 antibiotics listed by the European Food Safety Authority (EFSA) [20], namely, ampicillin, gentamicin, kanamycin, streptomycin, erythromycin, clindamycin, tetracycline, and chloramphenicol, and evaluated using the commercial E-test MIC Test Strip (Liofilchem, Roseto degli Abruzzi, Italy). The concentration on the strips ranged from 0.016 to 256 ug/mL for all antibiotics, except streptomycin (0.064–1024 ug/mL). The MIC test strips were placed on the surface of the inoculated agar and incubated at 37 °C for 48 h. The minimal inhibitory concentrations (MICs) were interpreted as the point at which the ellipse intersected the strip, as described in the MIC Test Strip technical guide.

### 2.6. Determination of Hemolytic Activity and Biogenic Amine

The hemolytic activities of *L. fermentum* KGC1601, *E. coli* KCTC2441 and *S. aureus* NCTC10788 were determined by streaking the cultures on blood agar plates (KisanBio, Seoul, Korea) and incubating them at 37 °C overnight. *E. coli* KCTC2441 and *S. aureus* NCTC10788 were used as positive controls for hemolytic activity. The hemolytic activities of the three species were determined by observing the size of the clear zones around colonies.

To measure Biogenic amines (BAs), 1 mL of cell-free supernatants was mixed with 200 μL of saturated NaHCO_3_, 20 μL of 2 M NaOH, and 0.5 mL of dansyl chloride solution (10 mg/mL of acetone). The mixture was heated at 70 °C for 10 min to derivatize the BAs; then, 200 μL of proline (100 mg/mL of water) was added to the mixture and then incubated for 15 min at 25 °C in the dark. The final volume of the mixture was made up to 5 mL with acetonitrile, and the resulting mixture was filtered through a 0.45 μm syringe filter. BAs were analyzed on a high-performance liquid chromatography system (LC-NETI/ADC, Jasco, Macclesfield, UK) equipped with a C18 column (ANPEL Laboratory analysis, Shanghai, China, 4.6 mm × 250 mm C18 column) with a detection wavelength of 254 nm. The mobile phase consisted of aqueous acetonitrile solution (67:33 of water) flowing at a rate of 0.8 mL/min.

### 2.7. Acid Resistance and Bile Salt Tolerance Tests

For acid resistance test, after culturing the strain, the supernatant was removed by centrifugation, and only cell pellets were collected, which were washed twice with phosphate-buffered saline (PBS) buffer (pH 7.4), re-suspended, and inoculated at 10^8^ colony-forming unit CFU/mL in PBS buffer adjusted to pH 2.5. After exposure for 3 h at 37 °C in an incubator, the cell pellets were cultured in MRS agar medium and the number of bacteria was measured.

For bile salt tolerance tests, after removing the supernatant by centrifuging the strain culture medium, only cell pellets were collected and used; they were next washed twice with PBS buffer (pH 7.4), re-suspended, and inoculated at 10^8^ CFU/mL in PBS buffer (pH 7.4) with 0.1% Oxgall (Kis-anBio, Seoul, Korea). After exposure to 37 °C in an incubator for 3 h, cell pellets were cultured in MRS agar medium and the number of bacteria was measured.

### 2.8. Identification of the Effect of Bile Salt Tolerance Using Scanning Electron Microscopy

The effect of bile salt on morphology of *L. fermentum* KGC1601 was analyzed using scanning electron microscopy (SEM). *L. fermentum* KCTC3112 was served as a control. The bacterial cells after treatment with bile salt (0.1%, *w/v*) for 3 h were collected by centrifugation, washed with and resuspended in PBS (pH 6.5, 20 mM potassium phosphate buffer, 8.5 g/L NaCl). The washed cell suspension was mounted onto a poly-L-lysine-coated slide glass and fixed with 2.5% (*v/v*) glutaraldehyde (TAAB Laboratories Equipment Ltd., Berkshire, UK) in PBS at room temperature for 1 h. The slide glass was rinsed with PBS, and post-fixed with 10 g/L osmium tetroxide in PBS at room temperature for 30 min. The slide glass was rinsed with PBS, and the cells were dehydrated in an ethanol series (50%, 70%, 90%, and 99.5%, *v/v*; 10 min each). The final step (99.5% ethanol) was repeated four times. The fixed cells were critical-point dried using liquid CO_2_ (HCP-2, Hitachi, Tokyo, Japan), and the slide glass was mounted on a metallic stub for coating with a 10 nm layer of Au-Pd using a sputter coater (E-101, Hitachi, Tokyo, Japan). The coated cells were visualized by SEM (JSM-6301F, JEOL Ltd., Akishima, Tokyo, Japan) using a 10 kV acceleration voltage.

### 2.9. Adhesion to HT-29 Cells

The intestinal adhesion ability of the strains was evaluated using HT-29 cells (a human colorectal adenocarcinoma cell line). *L. fermentum* KCTC3112 and *L. fermentum* IDCC3901 served as a control. The adhesion assay was performed using a modified version of Kim’s method [10]. Briefly, HT-29 cells (4 × 10^5^ cells/mL) were aliquoted into 12-well plates and incubated in RPMI medium without fetal bovine serum (FBS) and antibiotics at 37 °C in a 5% CO_2_ atmosphere overnight. Each strain (1 × 10^7^ CFU/mL) was inoculated into HT-29 cells and incubated at 37 °C in a 5% CO_2_ atmosphere for 2 h. Non-adherent cells were removed, and the adherent cells were detached with trypsin–EDTA solution, washed with PBS (pH 7.0), and then cultured on MRS agar plates. The following equation calculated the rates of adhesion of strains: adhesion (%) = (number of viable cells/initial number of cells) × 100.

### 2.10. Evaluation of the Anti-Inflammatory Potential

#### 2.10.1. Cell Culture

The murine macrophage cell line RAW 264.7 was cultured in high-glucose DMEM (WELGENE, Daejeon, Korea) supplemented with 10% heat-inactivated fetal bovine serum (FBS, WELGENE, Daejeon, Korea) and 1% penicillin/streptomycin (Gibco, Grand Island, NY, USA) at 37 °C under a humidified atmosphere of 5% CO_2_. Subcultures were performed when the cell confluency reached 80–90% at the plate.

#### 2.10.2. Quantitative Real-Time Polymerase Chain Reaction (qRT-PCR)

To evaluate the anti-inflammatory potential of the *L. fermentum* KGC1601, quantitative real-time polymerase chain reaction (qRT-PCR) analysis was performed. RAW 264.7 cells were seeded at the density of 7.5 × 10^5^ cells/well in a 12-well plate and incubated for 48 h. As the sample for the experiment, *L. fermentum* KGC1601-cultured media was used. Each sample was then added to the DMEM medium at concentrations of 250, 500 and 1000 μg/mL, and the plates were cultured continuously for 12 h. Next, lipopolysaccharide (LPS) was added to the medium at the concentration of 1 μg/mL to induce inflammation. The cells were incubated for 6 h under this condition before extracting the RNA.

The total RNA was collected by using the Hybrid-R RNA purification kit (GeneAll, Daejeon, Korea). The messenger RNA (mRNA) expression of the pro-inflammatory cytokines was analyzed by qRT-PCR. A sum of 10 μL of the 2X Prime Q-master Mix (GENET BIO, Daejeon, Korea), 3 μL mixture of the forward and reverse primer (10 pmol/μL), and 4 μL of DEPC-treated water were added to 3 μL of the cDNA diluted to 1/10. The qRT-PCR was performed using the AriaMx (Agilent, Palo Alto, CA, USA). The qRT-PCR process was performed in 3 steps, repeated for 40 cycles of denaturation at 95 °C for 20 s, annealing at 58 °C for 20 s, and elongation at 72 °C for 20 s. The primers of pro-inflammatory cytokines were used to perform qRT-PCR, as demonstrated in Table 1. The correction of the result value was performed using the mRNA expression level of glyceraldehyde 3-phosphate dehydrogenase (GAPDH).

#### 2.10.3. Enzyme-Linked Immunosorbent Assay (ELISA)

To examine whether pre-treatment of *L. fermentum* KGC1601-cultured media and the isolated EPS decrease the induction of cytokines by LPS, RAW 264.7 cells were seeded at the density of 7.5 × 10^5^ cells/well in a 12-well plate and incubated for 24 h. Each sample was pre-treated with the cells for 18 h; *L. fermentum* KGC1601-cultured media were treated with concentrations ranging from 250 to 1000 μg/mL, and EPS was treated with concentrations ranging from 100 to 1000 μg/mL. LPS was then added to the medium at the concentration of 1 mg/mL to induce inflammation. The cells were incubated for 18 h under this condition. The culture medium obtained from each well was centrifuged at 10,000 rpm for 3 min, and the supernatants were collected. Cytokines were quantified by ELISA MAX^TM^ Deluxe Set (BioLegend, SanDiego, CA, USA) according to the manufacturer’s recommendations. The cytokines in the culture medium were quantified as described above.

### 2.11. Statistics

In this study, all data were obtained from three independent experiments and presented with mean ± standard deviation. Statistical analysis was determined using unparied ANOVA, and significance was defined as * *p* < 0.05, ** *p* < 0.01, and *** *p* < 0.0001.

## 3. Results and Discussion

### 3.1. Identification of L. fermentum Isolates

**Figure 1:**
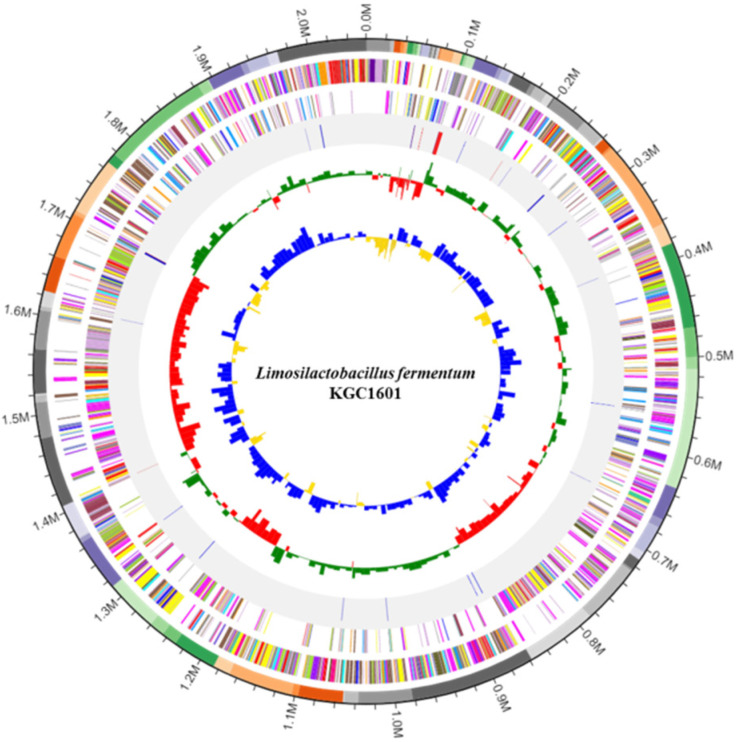
Genome map of *L. fermentum* KGC1601. From the outside to the center: 71 contigs, annotated reference genes (specifically, CDSs) found in the forward strand, in the reverse strand, rRNA, and tRNA were found in this genome, GC skew metric (higher-than-average values: green, lower-than-average values: red), and GC ratio metric (higher-than-average values: blue, lower-than-average values: yellow).

The isolates were identified by 16S rRNA gene sequencing and revealed >99% identity to *L. fermentum* upon BLAST analysis. As a result, the isolate was named *L. fermentum* KGC1601, and its general features and the circular genome map are depicted in Table 2 and Figure 1. The genome sequences were approximately 2.06 million base pairs in length (51.9% of the GC contents) and contained 1998 coding deoxyribonucleic acid (DNA) sequences (CDS), 11 rRNA genes, and 54 transfer RNA (tRNA) genes. In lactic acid bacteria, intrinsic resistance to several antibiotics was commonly noted, and these strains were reported to easily acquire antibiotic resistance and virulence factors [21]. These acquired genetic characteristics could be transferred to pathogens in the gastrointestinal system. Thus, in terms of safety assessment as a probiotic, it is essential to analyze the whole-genome sequencing as well as the acquired antibiotic resistance and virulence factors associated with the strain. According to the whole-genome analysis results, antibiotic resistance genes and virulence factors were not identified in the isolate *L. fermentum* KGC1601.

### 3.2. Carbohydrate Fermentation Patterns

The carbohydrate fermentation patterns of *L. fermentum* KGC1601 were different from those of *L. fermentum* KCTC3112 (Table 3). Of the 49 carbohydrates, D-xylose, D-galactose, D-lactose, D-trehalose, D-raffinose, and potassium 5-ketogluconate displayed different patterns. For instance, *L. fermentum* KGC1601 fermented D-xylose, D-trehalose, D-raffinose, and potassium 5-ketogluconate fermented, whereas *L. fermentum* KCTC3112 did not. These characteristic fermentation patterns can be useful in the industrial field to optimize culture conditions for boosting productivity.

### 3.3. Antibiotic Resistance

If the microorganism has an antibiotic resistance gene, the antibiotic effect may not appear during bacterial infection, and antibiotic resistance gene transfer may occur in the intestine. Therefore, it is important to evaluate the safety of probiotics to confirm the antibiotic resistance characteristics of microorganisms.

For the in silico safety analysis, we analyzed the potential antibiotic resistance gene virulence factors and the mobile elements of *L. fermentum* KGC1601. On the basis of the BLASTn algorithm and the VFDB, no putative virulence gene was identified in *L. fermentum* KGC1601, and neither were any genes related to antibiotic resistance. Furthermore, mobile elements, such as transposes, genomic island, and prophage in the genome were analyzed to predict the possibility of antibiotic resistance gene transfer. However, these genes were meaningless as they were not potential antibiotic resistance genes. Next, the MICs of *L. fermentum* KGC1601 against eight antibiotics were analyzed to verify the safety of our isolate (Table 4). As per the results, *L. fermentum* KGC1601 was susceptible to all antibiotics tested, except for kanamycin and streptomycin. Most *Lactobacillus* sp. are intrinsically resistant to aminoglycosides, such as kanamycin and streptomycin [22]. Thus, the resistance against kanamycin and streptomycin seems to be an intrinsic trait of this strain. Indeed, several studies have shown that several *Lactobacillus* species, including some commercially used strains, exhibit relative resistance to aminoglycoside antibiotics, such as gentamicin, kanamycin, and streptomycin [17,23,24]. As a result, *L. fermentum* KGC1601 can be considered safe in terms of antibiotic resistance.

### 3.4. Hemolytic Activities and Biogenic Amine Production

The hemolytic activity is the ability to destroy red blood cells to release hemoglobin, usually caused by pathogenic bacteria [25]. The identification of the hemolytic activity of lactic acid bacteria is one of the important factors for assessing the safety of probiotics. α- hemolysis is a hemolytic property that forms green colonies on blood agar by reducing the methemoglobin of hemoglobin in red blood cells, and *E. coli* typically shows α- hemolysis. *S. aureus* shows β-hemolysis as a representative. β-hemolysis is a hemolytic property that forms transparent colonies on blood agar by destroying red blood cells, and *S. aureus* typically shows the β-hemolysis property. γ-hemolysis means that no hemolysis is observed in the blood agar.

**Figure 2:**
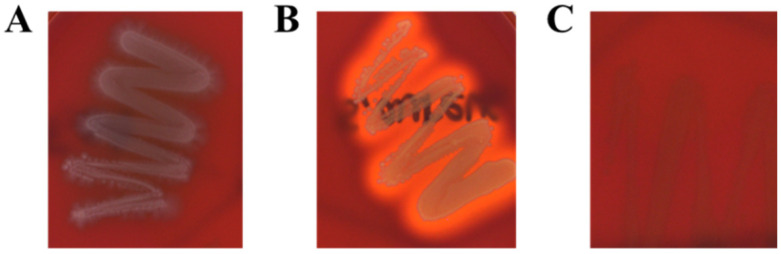
Hemolytic activities of *L. fermentum* KGC1601, *Escherichia coli KCTC2441*, and *Staphylococcus aureus NCTC10788*. *E. coli* were shown typical α-hemolysis (**A**) and *S. aureus* was shown typical β-hemolysis (**B**). However, *L. fermentum* KGC1601 (**C**) did not show any lysis of the blood cells.

In the present study, to determine the hemolytic activity of *L. fermentum* KGC1601, the hemolytic activity of *Escherichia coli* KCTC2441, and *Staphylococcus aureus* NCTC10788 was investigated as a positive control. While *E. coli* and *S. aureus* exhibited α- and β-hemolysis properties, respectively, *L. fermentum* KGC1601 did not exhibit any hemolytic activity on sheep blood agar (Figure 2).

Biogenic amines are the decarboxylation products of amino acids produced during the fermentation of lactic acid bacteria. Although biogenic amines are present in a variety of foods, they are also recognized as potential toxic factors for human health as they are the precursors to the formation of N-nitroso compounds, a well-known cancer-causing factor [26]. The toxicity of biogenic amines depends on their intake and the corresponding human sensitivity; small amounts of biogenic amines can be detoxified by intestinal amine oxidase [26,27]. On the other hand, large amounts of biogenic amines are commonly known to cause various symptoms such as nausea, vomiting, diarrhea, abdominal pain, rash, itching, headache, and high blood pressure [28]. In the supernatant of *L. fermentum* KGC1601 in this study, no biogenic amines including tyramine, histamine, putrescine, 2-phenethyamine, and cadaverine, were detected (data not shown).

### 3.5. Acid Resistance and Bile Salt Tolerance

For the ingested probiotics to reach the intestines and function properly, they must survive while passing through the human gastrointestinal tract. Thus, resistance to acid and bile salt is an essential characteristic of probiotics. The acid resistance and bile salt tolerance properties of this strain are shown in Table 5. When *L. fermentum* KGC1601 was cultured for 3 h in the MRS liquid medium adjusted to pH 2.5, the survival rate was approximately 66%, which is similar to that of the standard strain *L. fermentum* KCTC3112 under the same experimental conditions. Meanwhile, the bile salt tolerance of *L. fermentum* KGC1601 was superior to that of *L. fermentum* KCTC3112, the standard strain. *L. fermentum* KGC1601 cultured in a PBS buffer containing 0.2% bile salt for 3 h showed a survival rate of 76%. On the other hand, the standard strain *L. fermentum KCTC3112* was observed to survive by approximately 41% under the same experimental conditions.

**Figure 3:**
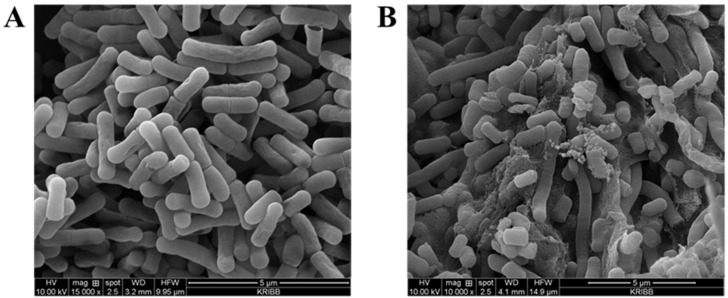
Effect of bile salt incubation of *L. fermentum* KGC1601 and KCTC 3112 for 3 h at 37 °C on cell morphology as studied under scanning electron microscope (20,000×). (**A**) *L. fermentum* KGC1601, (**B**) *L. fermentum* KCTC3112.

It was also confirmed through the SEM results that the *L. fermentum* KGC1601 exhibited excellent bile salt tolerance. The effect of bile salt after incubation of *L. fermentum* KGC1601 and *L. fermentum* KCTC3112 on its cell morphology was studied with SEM (Figure 3). *L. fermentum* KGC1601 incubated in PBS containing 0.1% bile salt showed a regular and smooth surface as shown in Figure 3A. Meanwhile, *L. fermentum* KCTC3112 incubated in the same condition exhibited severe membrane damage, consistent with disruption of the membrane integrity as shown in Figure 3B.

When lactic acid bacteria encounter the bile environment, how they adapt and survive and mechanisms of bile tolerance have been discussed through several studies [29]. Neutralization of bile toxicity through bile–efflux systems [30], cell protection through the activity of bile salt hydrolase [31], and changes in cell membrane structure and lipid composition [32,33] are related as a representative bile resistance mechanism of lactic acid bacteria. Among them, the excellent bile salt tolerance of *L. fermentum* KGC1601 can be explained by a mechanism related to the effect of bile on the cell-surface structures of lactic acid bacteria. Due to the lipophilic character of the bile structure, the bacterial cell membrane is one of the main targets of bile, destroying the structure of the bacterial envelope, which also affects the cell morphology [32,33]. Bile salt has a profound effect on the surface properties of lactic acid bacteria due to changes in cell membrane structure, lipid composition, and presence and properties of the external coat, which is associated with a cytoprotective effect, resulting in the enhancement in tolerance to bile. According to the results of this study (Table 5 and Figure 3), *L. fermentum* KGC1601 was observed to have a higher survival rate and a smoother cell surface in the bile environment compared to the *L. fermentum* KCTC3112, suggesting that this strain has an intrinsic bile tolerance or is highly adaptabile to the bile environment. Consequently, these results demonstrated that the bile salt tolerance properties of *L. fermentum* KGC1601 were significantly superior to those of *L. fermentum* KCTC 3112.

### 3.6. Adhesion Abilities

**Figure 4:**
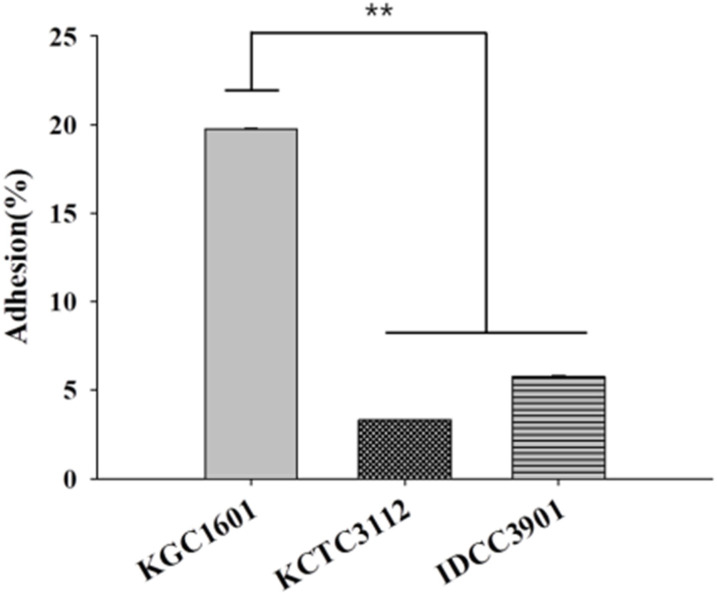
Adhesion ability of *L. fermentum* KGC1601, KCTC3112, and IDCC3901. Adhesion ability to HT-29 cells was measured. Data are presented as the mean ± standard deviation of values obtained from triplicate experiments (** *p* < 0.01). Adhesion (%) = (number of viable cells/initial number of cells) × 100.

Probiotics require sufficient interaction with the host to provide health benefits to the host gut [34]. Adhesion of the probiotics to the intestinal epithelial cells is essential for producing substances for growth and substances that inhibit pathogenic bacteria growth in the intestines [35,36]. Therefore, the ability to adhere to intestinal epithelial cells is one of the important properties as probiotics. In this study, the in vitro adhesion of *L. fermentum* strains to HT-29 cells was investigated. As shown in Figure 4, the adhesion rate of *L. fermentum* KGC1601 to HT-29 cells was 19.77%, whereas *L. fermentum* KCTC3112, and IDCC3901 strains had adhesion rates of 3.33% and 5.78, respectively. According to Kim et al., the adhesion ability of the two *L. fermentum* strains (MG4231 and MG4244) to HT-29 cells was at a level of 3–4% [10]. The results indicated that *L. fermentum* KGC1601 has a superior ability to adhere to HT-29 cells compared to the other two strains. Thus, the adhesion ability of *L. fermentum* KGC1601 to epithelial cells verifies its potential as a probiotic.

### 3.7. Anti-Inflammatory Activity

#### 3.7.1. Anti-Inflammatory Activity of *L. fermentum* KGC1601 on Raw 264.7 Cells by qRT-PCR

The inflammatory response is one of the multicascade defenses that protect the human body from exotic agents by removing the elimination process and initiating the recovery process. During this process, macrophages play an essential role in the recognition of threats from specific receptors. The present study results supported LPS-induced inflammatory actions by markedly increasing the expression of pro-inflammatory cytokines. The process of synthesis was through the activation of TNF-α, IL-1β, and IL-6 mRNA expression; excessive production of these cytokines is known to mediate numerous inflammatory diseases. Therefore, reduction in the expression of these cytokines is necessary for alleviating inflammatory disorders.

**Figure 5:**
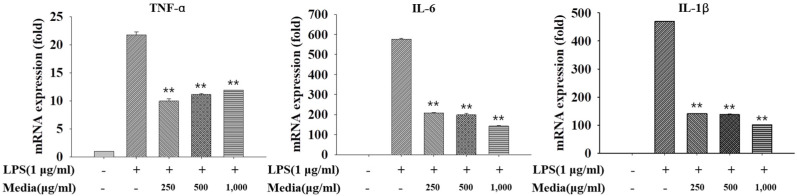
The mRNA expression levels of TNF-α, IL-1β, and IL-6, stimulated by the treatment with *L. fermentum* KGC1601-cultured media in RAW 264.7 cells through qRT-PCR. Data are presented as the mean ± standard deviation of values obtained from triplicate experiments. ** *p* < 0.01 versus the LPS-treated group.

To investigate the anti-inflammatory ability of *L. fermentum* KGC1601, cytokines generated by RAW 264.7 cells were measured with qRT-PCR analysis. The RAW 264.7 cells were pre-treated with *L. fermentum* KGC1601-cultured media as described previously, albeit with some minor modifications (250–1000 μg/mL). The inflammatory cytokines, including TNF-α, IL-1β, and IL-6, were measured. The relative gene expression levels, stimulated by the treatment with *L. fermentum* KGC1601-cultured media in RAW 264.7 cells, are presented in Figure 5.

When the pro-inflammatory activity by LPS (1 μg/mL) was treated in RAW 264.7 cells, the mRNA expression levels of TNF-α, IL-1β, and IL-6 were severely increased. In contrast, RAW 264.7 cells with different concentrations of *L. fermentum* KGC1601-cultured media were treated with LPS, the mRNA expression levels of the three cytokines were markedly down-regulated. In terms of IL-1β and IL-6, the expression levels were decreased in a dose-dependent manner according to the administration of *L. fermentum* KGC1601-cultured media, which suggests the anti-inflammatory ability of the *L. fermentum* strain, extraordinarily at the concentration of 1 mg/mL. Meanwhile, TNF-α exhibited the highest anti-inflammatory ability at a concentration of 250 μg/mL. As a result, it can be inferred that *L. fermentum* KGC1601-cultured media induces anti-inflammatory effects by regulating the expression of inflammatory cytokines. These results are consistent with the results of a study that found *L. fermentum* strains exhibit immunomodulatory-related functions. According to a study by Archer et al., *L. fermentum* MCC 2760 obtained from curd down-regulated the expression of pro-inflammatory cytokines such as IL-6, IL-1β and TNF-α in LPS-challenged Caco-2 cells, whereas the expression of interleukin-10 (IL-10) increased [37]. Although immunomodulatory properties of *L. fermentum* are strain-dependent, this study demonstrated that the *L. fermentum* KGC1601 isolated from ginseng exhibits anti-inflammatory effects.

#### 3.7.2. Anti-Inflammatory Activity of *L. fermentum* KGC1601 on Raw 264.7 Cells by ELISA

To confirm which component of the *L. fermentum* KGC1601-cultured media exhibits the anti-inflammatory effect, we focused on the EPS. EPS is one of the metabolites secreted by probiotic bacteria, and EPS isolated from various strains has been reported to have many beneficial properties such as antioxidant activity [38], immunomodulatory activity [39,40], and cholesterol-lowering effect [41]. In particular, several studies have been performed to evaluate the inflammatory response induced by EPS, produced by probiotic bacteria, in macrophage cell lines. According to Sungur et al., EPS from *Lactobacillus gasseri* isolated from a healthy human vagina exhibited anti-inflammatory activity with decreased TNF-α production and increased IL-10 production, respectively, in Hela cells [42]. Ciszek-Lenda et al. found that EPS produced by *Lactobacillus rhamnosus* KL37 modulates the immune response by stimulating the production of both pro- and anti-inflammatory cytokines in mouse peritoneal macrophages [43]. Based on these recent findings, in this study, EPS was isolated from *L. fermentum* KGC1601 as a candidate substance, and the anti-inflammatory effect of EPS in LPS-stimulated RAW 264.7 cells was investigated.

**Figure 6:**
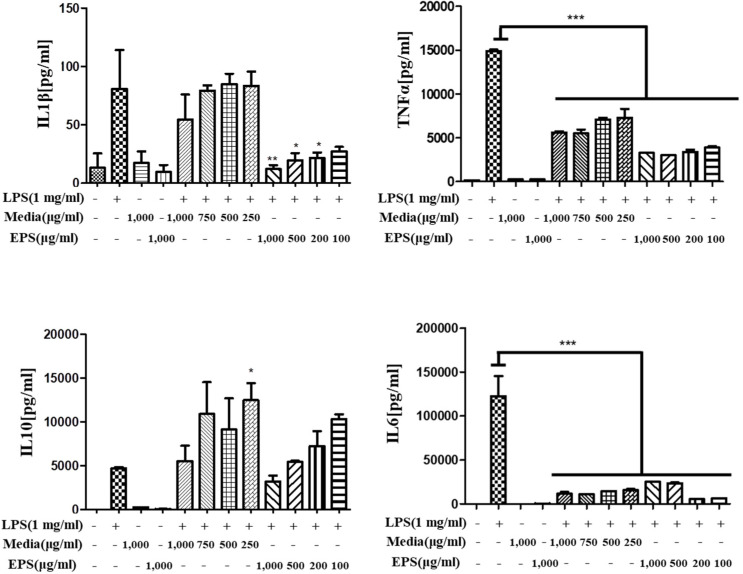
The expression levels of cytokines, induced by the treatment with *L. fermentum* KGC1601 in RAW 264.7 cells. Cytokines were quantified using ELISA. The four cytokines used for the experiment were TNF-α, IL-1β, IL-6, and IL-10. Cells were pre-treated with *L. fermentum* KGC1601-cultured media and isolated EPS. EPS inhibited the LPS-induced inflammatory response in RAW 264.7 cells. * *p* < 0.05, ** *p* < 0.01, and *** *p* < 0.0001 versus the LPS-treated group.

To confirm whether the pre-treatment of *L. fermentum* KGC1601 decreased the induction of cytokines by LPS, cytokines were quantified by the ELISA method. The Raw 264.7 cells were treated with *L. fermentum* KGC1601-cultured media and the isolated EPS as described previously, albeit with some minor modifications (250–1000 μg/mL). The inflammatory cytokines, including TNF-α, IL-1β, IL-6, and IL-10, were measured. The expression levels of cytokines measured by ELISA, stimulated by treatment with *L. fermentum* KGC1601-cultured media or the isolated EPS in RAW 264.7 cells, are presented in Figure 6.

When the LPS was treated in RAW 264.7 cells, the expression levels of four cytokines were increased compared to the control. In terms of TNF-α and IL-6, which are pro-inflammatory cytokines, the expression levels were significantly down-regulated in both the culture media and EPS pre-treatment group than in the only LPS-treatment group. Among the groups pre-treated with culture media and EPS at the same concentration, the expression levels of TNF-α and IL-6 were significantly decreased in the group pre-treated with EPS. The expression level of IL-1β was down-regulated only in the EPS pre-treatment group. These results indicate that EPS purified from *L. fermentum* KGC1601 contributes to the inhibition of the inflammatory response stimulated by LPS. Meanwhile, the expression level IL-10, one of the anti-inflammatory cytokines, showed a tendency to increase in the group pre-treated with the culture media compared the LPS-treatment-only group. In particular, the expression level of IL-10 was significantly increased in the group treated with the culture media at a concentration of 250 μg/mL.

The mechanism by which EPS derived from *L. fermentum* exhibits anti-inflammatory properties has not been specifically elucidated in this study. However, it can be clearly shown that the anti-inflammatory effect of this strain is due to the EPS when comparing the cytokine expression levels in RAW 264.7 cells pre-treated with culture media and EPS at the same concentration.

## 4. Conclusions

In this study, the characteristics and the safety profile of *L. fermentum* KGC1601 isolated from *panax ginseng* as a probiotic strain were confirmed. One of the advantageous properties of *L. fermentum* KGC1601 as a probiotic strain was that it has excellent bile salt tolerance, so it can be expected to have high stability while being ingested and passing through the digestive tract. In addition, one of the distinguishing properties of this strain was its anti-inflammatory effect, which showed that it has potential as a functional probiotic. By examining the expression level of cytokines related to inflammation regulation, it was confirmed that EPS purified from the culture media as well as the culture media of the *L. fermentum* KGC1601 had an anti-inflammatory effect. As a result, it was found that *L. fermentum* KGC1601 can be safely used as a potential anti-inflammatory probiotic agent for improving human health.

## 5. Patents

This section is not mandatory but may be added if there are patents resulting from the work reported in this manuscript.

## Figures and Tables

**Table 1:** Inflammatory cytokine primers for qRT-PCR.

Gene		Primer Sequence (5′ to 3′)	Product Size (bp)
IL-1β	Forward	AGG TCA AAG GTT TGG AAG CA	129
	Reverse	TGA AGC AGC TAT GGC AAC TG	
TNF-α	Forward	AGG GTC TGG GCC ATA GAA CT	103
	Reverse	CCA CCA CGC TCT TCT GTC TAC	
IL-6	Forward	GTC CTT CAG AGA GAT ACA GAA ACT	113
	Reverse	AGC TTA TCT GTT AGG AGA GCA TTG	
GAPDH	Forward	CCA TGG AGA AGG CTG GGG	159
	Reverse	CAA AGT TGT CAT GGA TGA CC	

**Table 2:** Genomic characteristics of *L. fermentum* KGC1601.

Title 1	Title 3
Identification	*Limosilactobacillus fermentum*
Genome size (bp)	2,058,202
GC contents (%)	51.9
CDS	1998
No. of rRNA genes	11
No. of tRNA genes	54

**Table 3:** Carbohydrates fermentation patterns of *L. fermentum* KGC1601 and KCTC3112.

Carbohydrates	Strains		Carbohydrates	Strains	
	KGC 1601	KCTC 3112		KGC 1601	KCTC 3112
Glycerol	−	−	Salicin	−	−
Erythritol	−	−	D-cellobiose	−	−
D-arabinose	−	−	D-maltose	+	+
L-arabinose	−	−	D-lactose	−	+
D-ribose	+	+	D-melibiose	+	+
D-xylose	+	−	D-saccharose(sucrose)	+	+
L-xylose	−	−	D-trehalose	+	−
D-adonitol	−	−	Inulin	−	−
Methyl-tolee(sucrose). o	−	−	D-melezitose	−	−
D-galactose	−	+	D-raffinose	+	−
D-glucose	+	+	Amidon (starch)	−	−
D-fructose	+	+	Glycogen	−	−
D-mannose	+	+	Xylitol	−	−
L-sorbose	−	−	Gentiobiose	−	−
L-rhamnose	−	−	D-turanose	−	−
Dulcitol	−	−	D-lyxose	−	−
Inocitol	−	−	D-tagatose	−	−
D-mannitol	−	−	D-fucose	−	−
D-sorbitol	−	−	L-fucose	−	−
Methylseolerch)crose). o	−	−	D-arabitol	−	−
Methylitolerch)crose). of	−	−	L-arabitol	−	−
N-acetylglucosamine	−	−	Potassium gluconate	−	−
Amygdalin	−	−	Potassium 2-ketogluconate	−	−
Arbutin	−	−	Potassium 5-ketogluconate	+	−
Esculin ferric citrate	+	+			

−: not utilized; +: strongly utilized.

**Table 4:** Minimal inhibitory concentrations (MICs) of *L. fermentum* KGC1601.

Antibiotic ^1^	AMP	GEN	KAN	STR	ERY	CLI	TET	CHL
Cut-off value (mg/L) ^2^	2	16	32	64	1	1	8	4
Observed MICs	0.016–0.023	12–16	>256	96–128	0.19–0.25	0.032	0.25–0.38	4
Assessment	S ^3^	S	R ^4^	R	S	S	S	S

^1^ AMP, ampicillin; GEN, gentamycin; KAN, kanamycin; STR, streptomycin; ERY, erythromycin; CLI, clindamycin; TET, tetracycline; CHL, chloramphenicol. ^2^ EFSA (European Food Safety Authority) Guidelines, 2018. ^3^ S, Susceptible. ^4^ R, Resistant.

**Table 5:** Acid resistance and bile salt tolerance of *L. fermentum* KGC1601 and KCTC3112.

Characteristics		Survival Rate (%)	
		KGC1601	KCTC3112
Acid resistance	0 h (log CFU/mL)	8.33	8.39
	3 h (log CFU/mL)	5.46	5.55
	Survival rate (%)	66	66
Bile salt tolerance	0 h (log CFU/mL)	8.33	8.39
	3 h (log CFU/mL)	6.35	3.44
	Survival rate (%)	76	41

## Data Availability

The data presented in this study are available on request from the corresponding author. The data are not publicly available due to privacy.

## References

[1] Gail C.M. Use of probiotics: Benefits of a balanced microbiome in the intestinal tract. Proceedings of the National Parent Club Canine Health Conference.

[2] Food and Agriculture Organization of the United Nations (FAO)/World Health Organization (WHO) (2006). Probiotics in Food: Health and Nutritional Properties and Guidelines for Evaluation.

[3] de Vrese M., Stegelmann A., Richter B., Fenselau S., Laue C., Schrezenmeir J. (2001). Probiotics—Compensation for lactase insufficiency. Am. J. Clin. Nutr..

[4] Golinska P., Wypij M., Agarkar G., Rathod D., Dahm H., Rai M. (2015). Endophytic actinobacteria of medicinal plants: Diversity and bioactivity. Antonie Leeuwenhoek.

[5] Nalini M.S., Prakash H.S. (2017). Diversity and bioprospecting of actinomycete endophytes from the medicinal plants. Lett. Appl. Microbiol..

[6] Goodwin P.H. (2022). The Endosphere Microbiome of Ginseng. Plants.

[7] Hong C.E., Kim J.U., Lee J.W., Bang K.H., Jo I.H. (2019). Metagenomic analysis of bacterial endophyte community structure and functions in *Panax ginseng* at different ages. 3 Biothech.

[8] Chowdhury E.K., Jeon J., Rim S.O., Park Y.-H., Lee S.K., Bae H. (2017). Composition, diversity and bioactivity of culturable bacterial endophytes in mountain-cultivated ginseng in Korea. Sci. Rep..

[9] Mo S.J., Nam B., Bae C.H., Park S.D., Shim J.J., Lee J.L. (2021). Characterization of novel *Lactobacillus paracasei* HY7017 capable of improving physiological properties and immune enhancing effects using red ginseng extract. Fermentation.

[10] Kim S., Choi S.I., Jang M., Jeong Y., Kang C.H., Kim G.H. (2020). Anti-adipogenic effect of *Lactobacillus fermentum* MG4231 and MG4244 through AMPK pathway in 3T3-L1 preadipocytes. Food Sci. Biotechnol..

[11] Zhu K., Tan F., Mu J., Yi R., Zhou X., Zhao X. (2019). Anti-obesity effects of *Lactobacillus fermentum* CQPC05 isolated from Sichuan pickle in high-fat diet-induced obese mice through PPAR-α signaling pathway. Microorganisms.

[12] Pan D.D., Zeng X.Q., Yan Y.T. (2011). Characterisation of *Lactobacillus fermentum* SM-7 isolated from koumiss, a potential probiotic bacterium with cholesterol-lowering effects. J. Sci. Food Agric..

[13] Zhao Y., Hong K., Zhao J., Zhang H., Zhai Q., Chen W. (2019). *Lactobacillus fermentum* and its potential immunomodulatory properties. J. Funct. Foods.

[14] Shih R.H., Wang C.Y., Yang C.M. (2015). NF-kappaB signaling pathways in neurological inflammation: A mini review. Front. Mol. Neurosci..

[15] Medzhitov R. (2008). Origin and physiological roles of inflammation. Nature.

[16] Rubin D.C., Shaker A., Levin M.S. (2012). Chronic intestinal inflammation: Inflammatory bowel disease and colitis-associated colon cancer. Front. Immunol..

[17] Yang S.Y., Chae S.A., Bang W.Y., Lee M., Ban O., Kim S.J., Jung Y.H., Yang J. (2021). Anti-inflammatory potential of *Lactiplantibacillus plantarum* IDCC 3501 and its safety evaluation. Braz. J. Microbiol..

[18] Kim K.T., Kim J.W., Kim S.I., Kim S., Nguyen T.H., Kang C.H. (2021). Antioxidant and anti-inflammatory effect and probiotic properties of lactic acid bacteria isolated from Canine and Feline feces. Microorganisms.

[19] Rychen G., Aquilina G., Azimonti G., Bampidis V., Bastos M.D.L., Bories G., Chesson A., Cocconcelli P.S., Flachowsky G., EFSA Panel on Additives and Products or Substances used in Animal Feed (FEEDAP) (2018). Guidance on the characterisation of microorganisms used as feed additives or as production organisms. EFSA J..

[20] Kwon M., Lee J., Park S., Kwon O.H., Seo J., Roh S. (2020). Exopolysaccharide isolated from *Lactobacillus plantarum* L-14 has anti-inflammatory effects via the Toll-like receptor 4 pathway in LPS-induced RAW 264.7 cells. Int. J. Mol. Sci..

[21] Chen L., Yang J., Yu J., Yao Z., Sun L., Shen Y., Jin Q. (2005). VFDB: A reference database for bacterial virulence factors. Nucleic Acids Res..

[22] Abriouel H., Muñoz M.D.C.C., Lerma L.L., Montoro B.P., Bockelmann W., Pichner R., Kabsch J., Cho G.-S., Franz C.M.A.P., Gálvez A. (2015). New insights in antibiotic resistance of *Lactobacillus* species from fermented foods. Food Res. Int..

[23] Lee B.S., Ban O., Bang W.Y., Chae S.A., Oh S., Park C., Lee M., Kim S.J., Yang J., Jung Y.H. (2021). Safety assessment of *Lactobacillus reuteri* IDCC 3701 based on phenotypic and genomic analysis. Ann. Microbiol..

[24] Kim H., Kim J.S., Kim Y., Jeong Y., Kim J.E., Paek N.S., Kang C.H. (2020). Antioxidant and probiotic properties of *Lactobacilli* and *Bifidobacteria* of human origins. Biotechnol. Bioprocess Eng..

[25] Nakajima Y., Ishibashi J., Yukuhiro F., Asaoka A., Taylor D., Yamakawa M. (2003). Antibacterial activity and mechanism of action of tick defensin against Gram-positive bacteria. Biochim. Biophys. Acta Gen. Subj..

[26] Doeun D., Davaatseren M., Chung M.S. (2017). Biogenic amines in foods. Food Sci. Biotechnol..

[27] Ancin-Azpilicueta C., Gonzalez-Marco A., Jimenez-Moreno N. (2008). Current knowledge about the presence of amines in wine. Crit. Rev. Food Sci. Nutr..

[28] Shalaby A.R. (1996). Significance of biogenic amines to food safety and human health. Food Res. Int..

[29] Ruiz L., Margolles A., Sánchez B. (2013). Bile resistance mechanisms in *Lactobacillus* and *Bifidobacterium*. Front. Immunol..

[30] Piddock L.J. (2006). Clinically relevant chromosomally encoded multidrug resistance efflux pumps in bacteria. Clin. Microbiol. Rev..

[31] Jones B.V., Begley M., Hill C., Gahan C.G., Marchesi J.R. (2008). Functional and comparative metagenomic analysis of bile salt hydrolase activity in the human gut microbiome. Proc. Natl. Acad. Sci. USA.

[32] Šušković J., Kos B., Matošić S., Besendorfer V. (2000). The effect of bile salts on survival and morphology of a potential probiotic strain *Lactobacillus acidophilus* M92. World J. Microbiol. Biotechnol..

[33] Kurdi P., Kawanishi K., Mizutani K., Yokota A. (2006). Mechanism of growth inhibition by free bile acids in *lactobacilli* and *bifidobacteria*. J. Bacteriol..

[34] Gioacchini G., Rossi G., Carnevali O. (2017). Host-probiotic interaction: New insight into the role of the endocannabinoid system by in vivo and ex vivo approaches. Sci. Rep..

[35] Zommiti M., Connil N., Hamida J.B., Ferchichi M. (2017). Probiotic characteristics of *Lactobacillus curvatus* DN317, a strain isolated from chicken ceca. Probiotics Antimicrob. Proteins..

[36] Srisesharam S., Park H.S., Soundharrajan I., Kuppusamy P., Kim D.H., Jayraaj I.A., Lee K.D., Choi K.C. (2018). Evaluation of probiotic *Lactobacillus plantarum* against foodborne pathogens and its fermentation potential in improving *Lolium multiflorum* silage quality. 3 Biotech.

[37] Archer A.C., Kurrey N.K., Halami P.M. (2018). In vitro adhesion and anti-inflammatory properties of native *Lactobacillus fermentum* and *Lactobacillus delbrueckii* spp. J. Appl. Microbiol..

[38] Adesulu-Dahunsi A.T., Jeyaram K., Sanni A.I., Banwo K. (2018). Production of exopolysaccharide by strains of *Lactobacillus plantarum* YO175 and OF101 isolated from traditional fermented cereal beverage. Peer. J..

[39] Wu M.H., Pan T.M., Wu Y.J., Chang S.J., Chang M.S., Hu C.Y. (2010). Exopolysaccharide activities from probiotic *bifidobacterium*: Immunomodulatory effects (on J774A. 1 macrophages) and antimicrobial properties. Int. J. Food Microbiol..

[40] Górska S., Jachymek W., Rybka J., Strus M., Heczko P.B., Gamian A. (2010). Structural and immunochemical studies of neutral exopolysaccharide produced by *Lactobacillus johnsonii* 142. Carbohydr. Res..

[41] Salazar N., Neyrinck A.M., Bindels L.B., Druart C., Ruas-Madiedo P., Cani P.D., Reyes-Gavilán C.G., Delzenne N.M. (2019). Functional effects of EPS-producing *bifidobacterium* administration on energy metabolic alterations of diet-induced obese mice. Front. Immunol..

[42] Sungur T., Aslim B., Karaaslan C., Aktas B. (2017). Impact of Exopolysaccharides (EPSs) of *Lactobacillus gasseri* strains isolated from human vagina on cervical tumor cells (HeLa). Anaerobe.

[43] Ciszek-Lenda M., Nowak B., Śróttek M., Gamian A., Marcinkiewicz J. (2011). Immunoregulatory potential of exopolysaccharide from *Lactobacillus rhamnosus* KL37. Effects on the production of inflammatory mediators by mouse macrophages. Int. J. Exp. Pathol..

